# Hemorrhagic fever with renal syndrome caused by destruction of residential area of rodent in a construction site: epidemiological investigation

**DOI:** 10.1186/s12879-022-07744-1

**Published:** 2022-09-29

**Authors:** Xiao Wei, Biao Meng, Hong Peng, Yan Li, Min Liu, Hairui Si, Rui Wu, Hailong Chen, Ying Bai, Yan Li, Qunling Feng, Changjun Wang, Xiangna Zhao

**Affiliations:** 1 Centers for Disease Control and Prevention of PLA, Beijing, China; 2 Department of Epidemiology and Biostatistics, School of Public Health, Anhui Medical University, Hefei, China; 3 PLA 63750 Hospital, Xi’an, Shaanxi China; 4 Xi’an Center for Disease Control and Prevention, Xi’an, Shaanxi China

**Keywords:** Hemorrhagic fever with renal syndrome, Hantavirus, Hantaan virus

## Abstract

**Background:**

An outbreak of hemorrhagic fever with renal syndrome (HFRS), caused by a Hantavirus, affected nine adult males in the southwest area of Xi’an in November 2020 was analyzed in this study.

**Methods:**

Clinical and epidemiological data of HFRS patients in this outbreak were retrospectively analyzed. The whole genome of a hantavirus named 201120HV03xa (hv03xa for short) isolated from *Apodemus agrarius* captured in the construction site was sequenced and analyzed. In addition, nine HFRS patients were monitored for the IgG antibody against the HV N protein at 6 and 12 months, respectively.

**Results:**

In this study, inhalation of aerosolized excreta and contaminated food may be the main source of infection. Genome analysis and phylogenetic analysis showed that hv03xa is a reassortment strain of HTNV, having an S segment related to A16 of HTN 4, an M segment related to Q37 and Q10 of HTN 4, and an L segment related to prototype strain 76–118 of HTN 7. Potential recombination was detected in the S segment of hv03xa strain. The anti-HV-IgG level of all the patients persist for at least one year after infection.

**Conclusions:**

This report documented an HFRS outbreak in Xi’an, China, which provided the basic data for epidemiological surveillance of endemic HTNV infection and facilitated to predict disease risk and implement prevention measures.

**Supplementary Information:**

The online version contains supplementary material available at 10.1186/s12879-022-07744-1.

## Introduction

Hemorrhagic fever with renal syndrome (HFRS) is a group of clinically similar illnesses caused by hantaviruses (such as Hantaan virus, Dobrava virus, Seoul virus, and Puumala virus), each of which causes diseases with different severity [1]. Hantaviruses are emerging public health threat and widely distributed in eastern Asia and Europe [2, 3]. In China, 30,000–50,000 cases of HFRS are documented annually, accounting for > 90% of total numbers worldwide [4, 5], and Hantaan virus and Seoul virus are the two main pathogens [6]. Shaanxi province is one of the provinces with the most serious incidence of HFRS in China [7, 8]. In 2010, the number of HFRS cases in Shaanxi province ranked the top among all provinces in China, and the incidence of HFRS in Xi'an city accounts for more than 90% of Shaanxi province [6, 9].

Generally, symptoms of HFRS appear within 1–2 weeks of exposure to source of infection [10]. Early symptoms such as headache, back and abdominal pain, fever, chills, nausea and blurred vision develop suddenly, and later progress to acute kidney injury and severe fluid overload [10, 11]. Complete recovery will take weeks or months. The severity of the disease varies depending upon the virus causing the infection. Hantaan and Dobrava viruses show the highest mortality ranging from 5 to 10% [12].

Hantaviruses (Family *Hantaviridae*, Order *Bunyavirales*) are negative sense RNA viruses and consists of four proteins, RNA-dependent RNA polymerase (RdRp), two membrane glycoproteins (G_N_ and G_C_), and a nucleocapsid (N) protein. N protein is encoded by S fragment, and the main function is to wrap three fragments of viral RNA, and the protein has strong immunogenicity. Both Gn and Gc are membrane glycoproteins encoded by M fragment with neutralizing antigen sites and hemagglutination sites. Gc, with membrane-distal localization and Gn, with selective pressure of the humoral immune response, these two antigenic sites exist independently but may overlap partially. L segment encodes RNA-dependent-RNA polymerase, which plays an important role in viral replication [13, 14].

Rodents are the natural reservoir for hantaviruses [15]. Contact with rodent feces or aerosol particles formed after bites from infected rodents are the main routes of hantavirus transmission to humans [7]. 30 *Apodemus agrarius* were captured in the construction site after the outbreak and 4 of them were detected positive for HTNV. A HTNV strain named 201120HV03xa (hv03xa for short) was isolated in the *A. agrarius* and the sequence was analyzed.

Reassortment, recombination, and genetic drift confer the genetic diversity in RNA viruses in nature. Segmented RNA viruses preferentially give rise to genetic reassortment rather than recombination [16]. The hv03xa strain isolated in this study is likely a reassortment strain, having an S segment related to A16 of HTN 4, an M segment related to Q37 and Q10 of HTN 4, and an L segment related to prototype strain 76–118 of HTN 7 [17].

## Materials and methods

### Outbreak detection

**Fig. 1:**
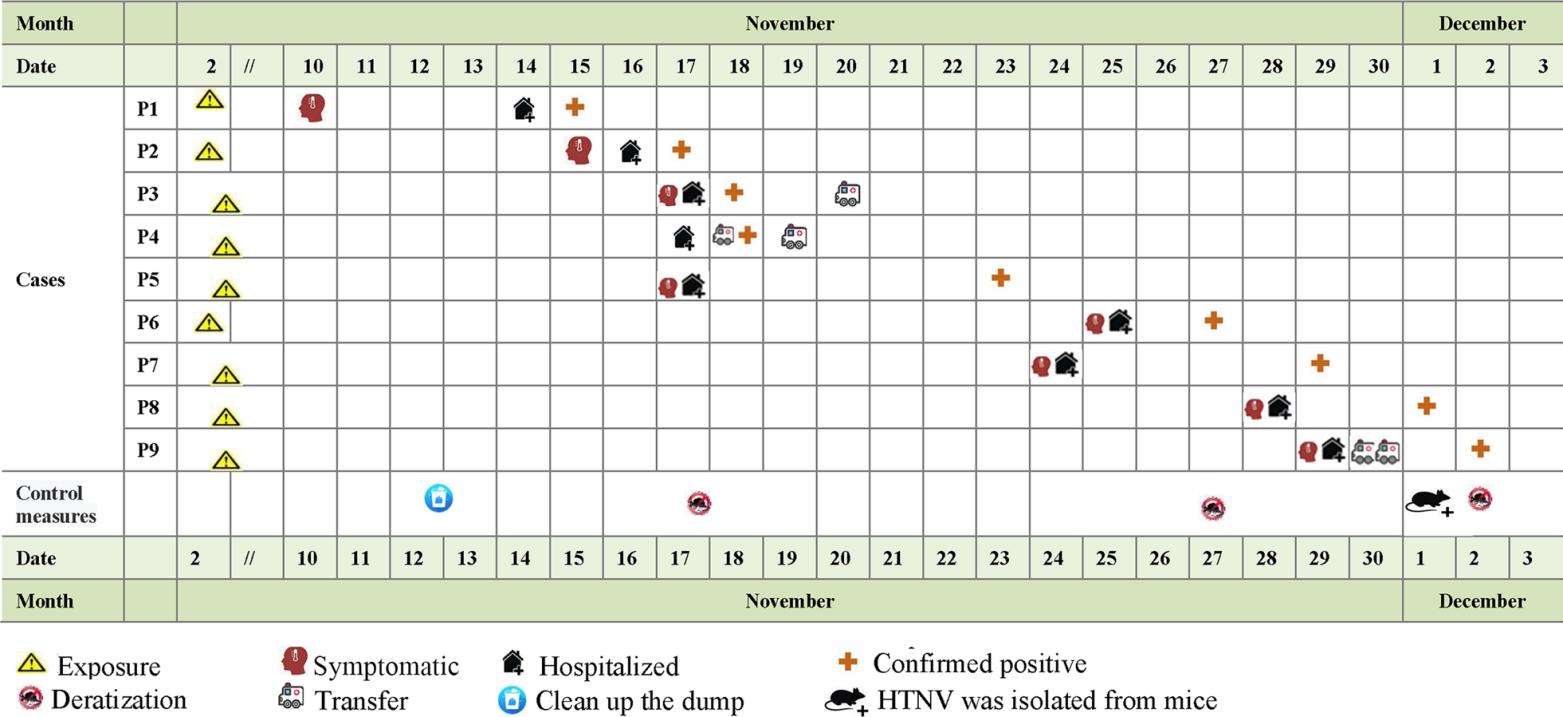
Timeline of relevant exposures and clinical symptoms of the 9 patients with HFRS

**Fig. 2:**
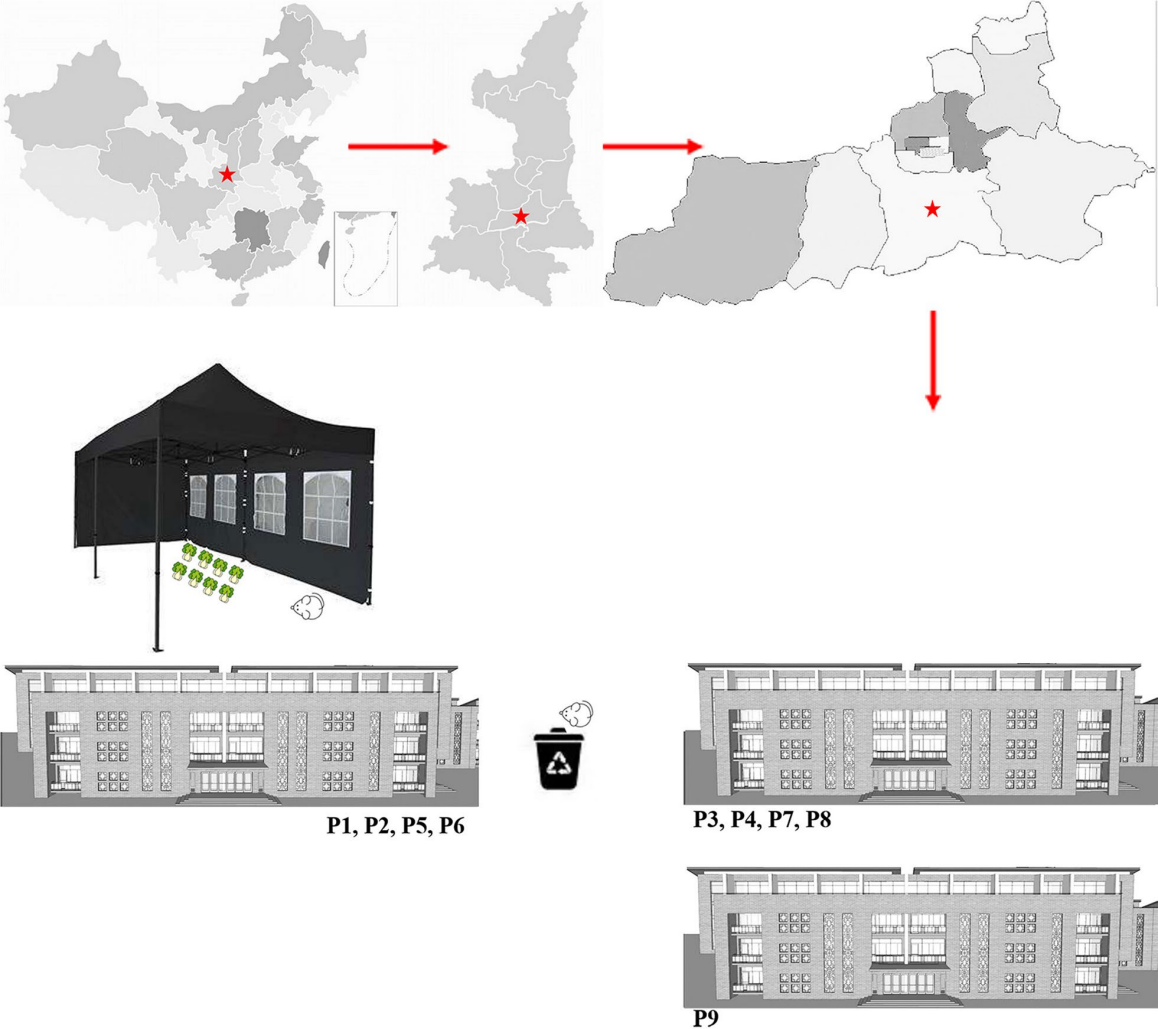
Geographical distribution of the nine confirmed HFRS patients at Chang’an district, Xi’an, China

On November 15th, 2020, No. 986 hospital received a positive case of Hantavirus and reported that there was a possible outbreak of HFRS. In the following 3 days, 3 more HFRS patients were confirmed. As of December 2, a total of nine patients had been diagnosed with HFRS (Fig. 1). The site of the outbreak is in a new construction site, the construction action destructed the rodent’s residence. Between the two rows of dormitories where the patients lived there was a garbage station. In the north of the dormitory there was a tent, piled with Chinese cabbages and other vegetables for daily consumption. Rat tracks were observed inside the tent and near the garbage station (Fig. 2). The patients were classified into mild, moderate, and severe based on clinical classification of HFRS [10].

### Case definition in outbreak

HFRS was diagnosed in all cases according to Enzyme-linked immunosorbent assay (ELISA)-based detection of anti-HV IgM in serum samples obtained from patients during acute phase of the disease [18]. The so-called acute phase of the disease is defined as the period of febrile, hypotensive, and oliguric phases [10, 18, 19]. In this study, ELISA was also used to detect the titers of serum IgG/M antibody over time in patients.

### Epidemiological investigation

An epidemiological investigation was carried out after confirmation of the HFRS outbreak. HFRS-like syndrome (fever, hypotensive, proteinuria, and acute renal failure), activity tracks, clinical symptoms, and medical records of febrile patients residing at the construction site were retrospectively collected. The Hantavirus IgG antibody levels of all patients were monitored.

### Measures taken to control HFRS expansion

Epidemic prevention measures were carried out immediately after the outbreak. Rodent capture equipment, including 200 sticky rat boards, 800 traps and 30 cages, was set up immediately after the occurrence of the index case. The delivery of outdoor anticoagulant rodenticide was finished on November 16th. The garbage station was cleaned on November 12th-13th, the vegetables were removed from the tents and final disinfection was performed on the sites. A customized mouse baffle is installed in the canteen, and closed storage of materials is adopted. The local CDC strengthened the canteen daily cleaning, tableware/kitchenware strict disinfection, the management of water sanitation, the quarantine of personnel and vehicles. By the end of December, 1268 people had been vaccinated with inactivated bivalent (HTN + SEO type) HFRS vaccine made from hamster kidney cell culture (Changchun Institute of Biological Products, China). Experts were invited to carry out disease knowledge education, provide guidance on prevention and control work, educate personnel to maintain hygiene habits, and avoid panic. The temperatures of all staff were measured daily to check for fever or other uncomfortable conditions, and medical treatment was arranged in time when abnormal conditions occurred. The epidemic was basically brought under control about December 6 and eliminated about December 15th.

### Isolation of Hantaan virus and genome sequencing

*A. agrarius* collected was sterilized with 75% alcohol, and the chest was dissected and a small lung tissue was taken out with a sterile scissors and tweezers. Appropriate amount of Trizol was added followed by tissue polishing. Magnetic bead method was used for nucleic acid extracting. HTNV-specific reverse transcription-polymerase chain reaction (RT-PCR) was conducted for viral RNA [20]. T25 VERO-E6 cells were cultured to a single layer, and the above-mentioned positive lung specimens were added to the cells for tiled adsorption [21]. Supernatant was removed and cell-sustaining fluid was added after 1.5 h. Viral CT values were detected after continuous cultivation of 21 days [22].

Viral RNA was extracted using RNeasy Plus Mini Kit (50) (QIAGEN 74134) [23]. Reverse transcription and amplification were performed using Ovation RNA-Seq System V2 (Tecan 7102-08). Amplification products were subjected to sequencing using Oxford Nanopore Gridion X5 instrument [24]. Library was constructed using ligation sequencing kit (SQK-LSK109) and the sequencing chip used in this study was FLO-MIN106D [25]. The consistency sequences were generated by mapping, with the known Hantaan virus whole genome as a reference sequence.

### Phylogenetic and recombination analysis

The nucleotide sequences of S, M, and L segments of hv03xa strain were determined from virus-infected lungs of *A. agrarius*. Sequences were aligned and compared with HTNV sequences available in GenBank using Mega (version 7.0) with default parameters [26]. For the phylogenetic analysis, the Maximum likelihood phylogenetic trees were generated, and topologies were evaluated by 1000 bootstrap replicates [26].

Alignments of Sequence sets to HTNV S, M, and L segments were analyzed using the Recombination Detection Program 4 (RDP4) software package [27]. RDP [28], GENECONV [29], BOOTSCAN [30], MaxChi [31], Chimaera [30], SISCAN [32] and 3SEQ [33] were used only on events with P values less than 0.05. The recombination event was confirmed if it satisfied by four or more methods when the P-value < 0.05 and the RDP recombination consensus score (RDPRCS) was > 0.6 [34]. The P-value < 0.05 with the RDPRCS between 0.4 and 0.6 indicated the possibility of the recombination event [34]. RDPRCS < 0.4 and P-value < 0.05 suggested the rejection of the recombination [34]. All parameters were left at the default settings of RDP4.

### Antibody response to HTNV in patients

With a goal of determining the dynamics and duration of immunity produced after HTNV infection, sera collection was made 6 months and 12 months post-symptom onset. The anti-HV-IgG was tested by ELISA (ZC-M6404, Shanghai zcibio technology Co., Ltd) according to the manufacturer’s instructions. Briefly, the test was carried out by adding diluted serum onto a HV nucleocapsid protein antigen coated plate. After incubation for 30 min at 37 °C, wells were washed and incubated with 100 μl mouse anti-human IgG antibody labelled with horseradish peroxidase for 30 min at 37 °C. The IgG antibody captured by the antigen was detected by measuring the optical density (OD 450 nm) after adding the stop solution (0.2 M sulfuric acid). Each sample was repeated three times.

### Ethics statement

All participants signed an informed consent form prior to entering the study. The study conformed to the ethical guidelines of the 1975 Declaration of Helsinki. All human and animal experimental protocols in this study were approved by the Ethics Committee of the PLA 63750 Hospital. All human and animal experimental procedures were carried out in accordance with relevant guidelines and regulations.

## Results

### Descriptive epidemiology

Nine HFRS patients developed symptoms consecutively from November 10th to 29th at Chang’an district, located in the southwest of Xi’an. All the patients were from the same construction site. The rodent residence in the surrounding place was destroyed because of the construction action. It is said that the rodent population density was significantly increased comparing with the same period in the past. Some Chinese cabbage was stored in a tent of their residence for their daily consumption (Fig. 2). On November 2nd, the index patient (a 22-year-old male) transported the cabbage from the tent to the kitchen with two other patients (P 2 and P6). They saw rats in the tent and in the garbage station near the tent (less than 50 m). The index patient developed a headache (November 10th) eight days after transporting the cabbage and vomited on the evening of November 13th. On the morning of November 14th, he developed a fever (39 °C) and was admitted to the hospital. Nucleic acid tests confirmed that he was not infected with COVID-19. On November 15th, he was diagnosed with hemorrhagic fever with renal syndrome (HFRS) and classified as moderate patient. P2 and P6 became ill on November 15th and November 25th, respectively. P3, P4, P7, and P8 lived in a dormitory near the garbage station (Fig. 2), and they frequently took out garbage to the garbage station before the onset of illness. P5 and P9 frequently visited the tent before they had symptoms. The timeline of disease prevalence was presented in Fig. 1.

The clinical characteristics of the patients were summarized in Additional file 1: Table S1. Based upon clinical classification of HFRS [10], of the 9 patients, 2 had no bilateral renal abnormalities, no hemorrhagic spots, and no bulbar conjunctival congestion, they were classified as mild. Four patients experienced moderate illness, they presented with symptoms such as oliguria, bulbar conjunctival congestion, and subcapsular effusion of both kidneys. Three patients presented with oliguria, bilateral bulbar conjunctival hyperemia and edema, visible hemorrhagic spots, hypotension, bilateral kidney pain, bilateral subcapsular effusion and other symptoms, they were defined as severe patients.

### The possible source of the outbreak

The index case transported Chinese cabbage that may have been contaminated by the rodents lived inside or outside the tent. One week later, the index case presented with symptoms like HFRS. We cannot directly prove that the rodents visited the tent led to this outbreak; however, there was a probable association. First, the patients (P2 and P6) who transported Chinese cabbage with the index patient presented with symptoms afterwards. Second, the construction action caused the destruction of rodent residence and their migration to the workers’ residence (the food storage tent, garbage station, etc.). Traces of rodents were also observed in areas where the patients had been visited. The contact between humans with contaminated vegetables in the tent and aerosolized excrement (saliva, urine, and feces) in the garbage station greatly increased the risk of infection. Third, a Hantavirus strain was isolated from the lung tissue in *A. agrarius* captured in the construction site.

### Control measures

After confirmation of the outbreak on November 15th, demic prevention guarantee initiated a rodent eliminate program. Rodent capture was performed in the surrounding place. Meantime, publicity education efforts was also taken to control HFRS expansion. After that, the rodent density dropped obviously after rodent control efforts. Correspondingly, patient counts dramatically declined after December 1, approximately 10 days after the implementation of rodent control.

### Phylogenetic analysis of HTNV

The full-length of S, M, L segments from a hantavirus strain (named 201120HV03xa, hv03xa for short) isolated from *A. agrarius* was sequenced and analyzed. The GenBank accession number for the S, M, L segment of 201120HV03xa reported in this paper is ON661335, OP094683, ON661337, respectively. The full-length S segment of hv03xa strain is 1696 nucleotides, with a predicted nucleocapsid protein of 429 amino acids. The entire M segment of hv03xa strain is 3615 nucleotides, encoding a predicted Gn/Gc envelope glycoproteins with functional roles in the viral escape from immunological responses. The glycoprotein precursor contains 1,135 amino acids with the highly conserved pentapeptide WAASA motif being found at amino acid positions 644–648. The full-length of L segment from hv03xa strain is 6,533 nucleotides, with a predicted RdRp (RNA-dependent RNA polymerase) of 2,151 amino acids, starting at nucleotide position 38 and including 43 nucleotides of the 3’ noncoding region.

**Fig. 3:**
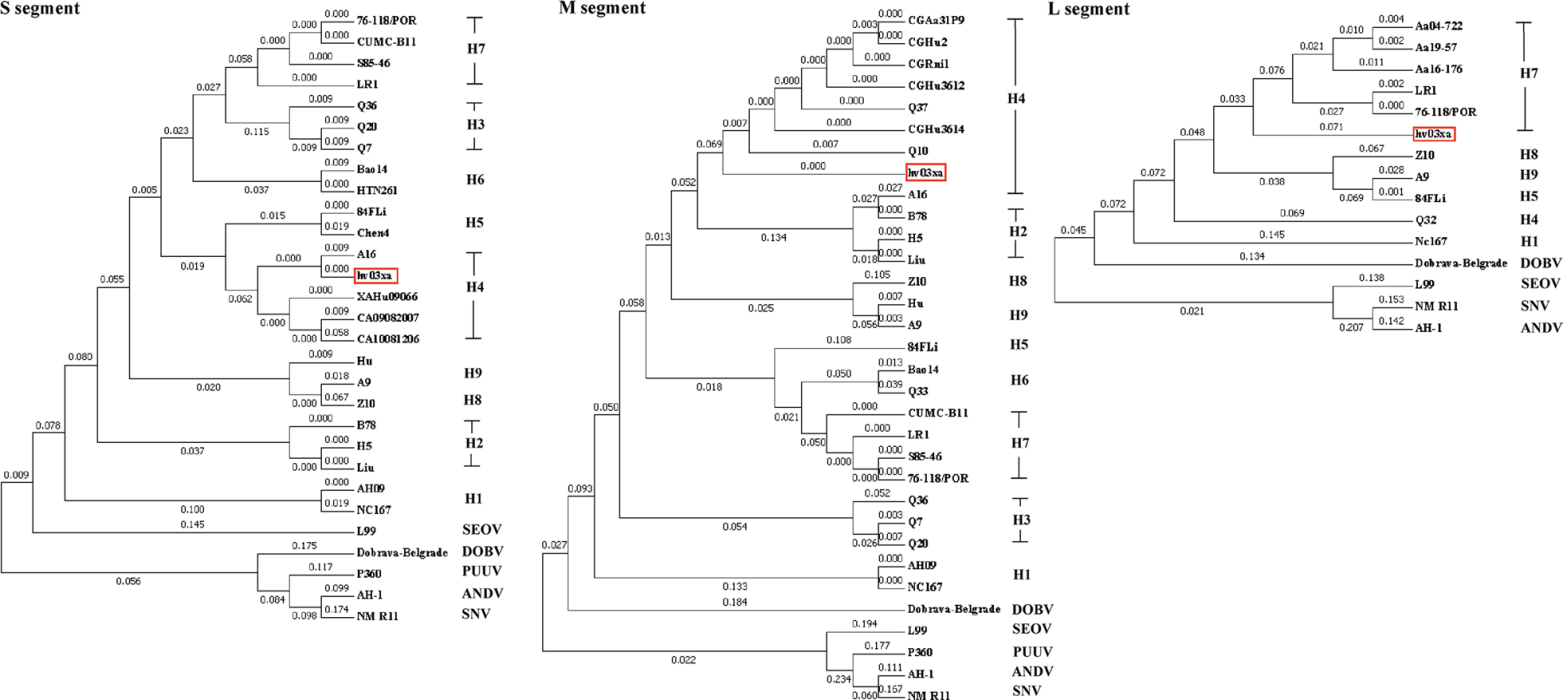
Phylogenetic trees for hantaviruses based on the sequences of the S, M, L segments at nucleotide level. The identifiers on the right of the figure indicated the subtype of HTNV and the reference viruses, including DOBV (Dobrava/Belgrade virus), SEOV (Seoul virus), PUUV (Puumala virus), ANDV (Andes hantavirus), and SNV (Sin Nombre virus)

Strains information for sequence analysis in this study were shown in Table 1. The branches of the phylogenetic tree of Hantaan virus formed nine HTN clades (designated subtypes HTN 1–9) [17]. Phylogenetic analysis indicated that the hv03xa strain is likely a reassortment strain of HTNV. S segment nucleotide sequence of hv03xa strain was closely related to isolates of subtype HTN 4, including Xi’an isolates XAHu09066, CA09082007, CA10081206 and Shanxi isolate A16 [7], with identity of 98.94%, 98.88%, 98.53% and 98.70%, respectively. M segment of hv03xa strain was closely related to Guizhou strains Q37, Q10, CGHu3614, CGHu3612, CGRni1, CGAa31P9 and CGHu2 of subtype HTN 4 [17], with identity of 99.33%, 98.67%, 98.20%, 98.09%, 98.06%, 92.93% and 92.79%, respectively. L segment of hv03xa strain was closely related with strains originated from South Korea, including 76–118/PRO (84.27% identity), Aa04-722 (84.64% identity), Aa19-57 (84.63% identity) and Aa16-176 (84.62% identity), which were in the subtype HTN 7 clade (Fig. 3).

**Table 1:** Strains information for sequence analysis in this study

Group	Strains	Accession no			Host	Country	Province
		S	M	L			
HTN Viruses							
HTNV 1	AH09	AF285264	AF285265	–	Niviventer niviventer	China	Anhui
	NC167	AB027523	AB027115	DQ989237	Niviventer niviventer	China	Anhui
HTNV 2	B78	AB027093	AB027056	–	Homo sapiens	China	Shandong
	H5	AB127996	AB127993	–	Homo sapiens	China	Heilongjiang
	Liu	AF288649	AF288648	–	Homo sapiens	China	Shandong
HTNV 3	Q7	AB027095	AB027058	–	Apodemus agrarius	China	Guizhou
	Q20	AB027096	AB027059	–	Apodemus agrarius	China	Guizhou
	Q36	AB027094	AB027057	–	Apodemus agrarius	China	Guizhou
HTNV 4	A16	AB027099	AB027063	–	Apodemus agrarius	China	Shanxi
	XAHu09066	JF421284	–	–	Homo sapiens	China	Xi'an
	CA09082007	HQ834499	–	–	Apodemus agrarius	China	Xi'an
	CA10081206	HQ834503	–	–	Apodemus agrarius	China	Xi'an
	CGAa31P9	–	EF990924	–	Apodemus agrarius	China	Guizhou
	CGHu2	–	EU363819	–	Homo sapiens	China	Guizhou
	CGHu3612	–	EF990923	–	Homo sapiens	China	Guizhou
	CGHu3614	–	EF990922	–	Homo sapiens	China	Guizhou
	CGRni1	–	EU363815	–	Rattus nitidus	China	Guizhou
	Q10	–	AB027062	–	Apodemus agrarius	China	Guizhou
	Q32			DQ371906	Apodemus agrarius	China	Guizhou
	Q37	–	AB027064	–	Apodemus agrarius	China	Guizhou
HTNV 5	84FLi	AF366568	AF366569	AF336826	Homo sapiens	China	Shanxi
	Chen4	AB027101	–	–	Homo sapiens	China	Anhui
HTNV 6	Bao14	AB127998	AB127995	–	Apodemus agrarius	China	Heilongjiang
	HTN261	AF252259	–	–	–	China	Heilongjiang
	Q33		AB027065		Apodemus agrarius	China	Guizhou
HTNV 7	76-118/PRO	KT885049	KT885048	KT885047	Apodemus agrarius	South Korea	–
	CUMC-B11	U37768	U38177	–	–	South Korea	–
	S85-46	AF288659	AF288658	–	Crocidura russula	China	Sichuan
	LR1	AF288294	AF288293	AF288292	Apodemus agrarius	China	–
	Aa04-722	–	–	KU207174	Apodemus agrarius	South Korea	–
	Aa19-57	–	–	MW796149	Apodemus agrarius	South Korea	–
	Aa16-176	–	–	MH598473	Apodemus agrarius	South Korea	–
HTNV 8	Z10	NC_006433	NC_006437	NC_006435	Homo sapiens	China	Zhejiang
HTNV 9	A9	AF329390	U00150	AF293665	Apodemus agrarius	China	Jiangsu
	Hu	AB027111	AB027077	–	Homo sapiens	China	Hubei
Other HTN virus strains of undetermined subtype							
	TJJ16	AY839871			Rattus confucianus	China	Tianjin
	CGAa75	EU092220			Apodemus agrarius	China	Guizhou
	CGRn53	EF990907			Rattus norvegicus	China	Guizhou
	CGRn93MP8	EF990905			Rattus norvegicus	China	Guizhou
	YU61	AY748308				China	
	YU62	AY748309				China	
	KY	GU140098			White rat	China	Yunnan
	Z37	AF187082				China	Zhejiang
Reference strains							
ANDV	AH-1	AF324902	AF324901	AF324900	–	Argentina	–
PUUV	P360	L11347	L08755	–	Clethrionomys glareolus	Russia	–
SEOV	L99	AF288299	AF288298	AF288297	Rattus losea	China	Jiangxi
	ZT10	AY766368				China	Zhejiang
DOBV	Dobrava-Belgrade	JQ026204	JQ026205	JQ026206	Apodemus flavicollis	Germany	–
SNV	NMR-11	L37904	L37903	L37902	Peromyscus maniculatus	The United States	–

### Recombination analysis of HTNV

**Fig. 4:**
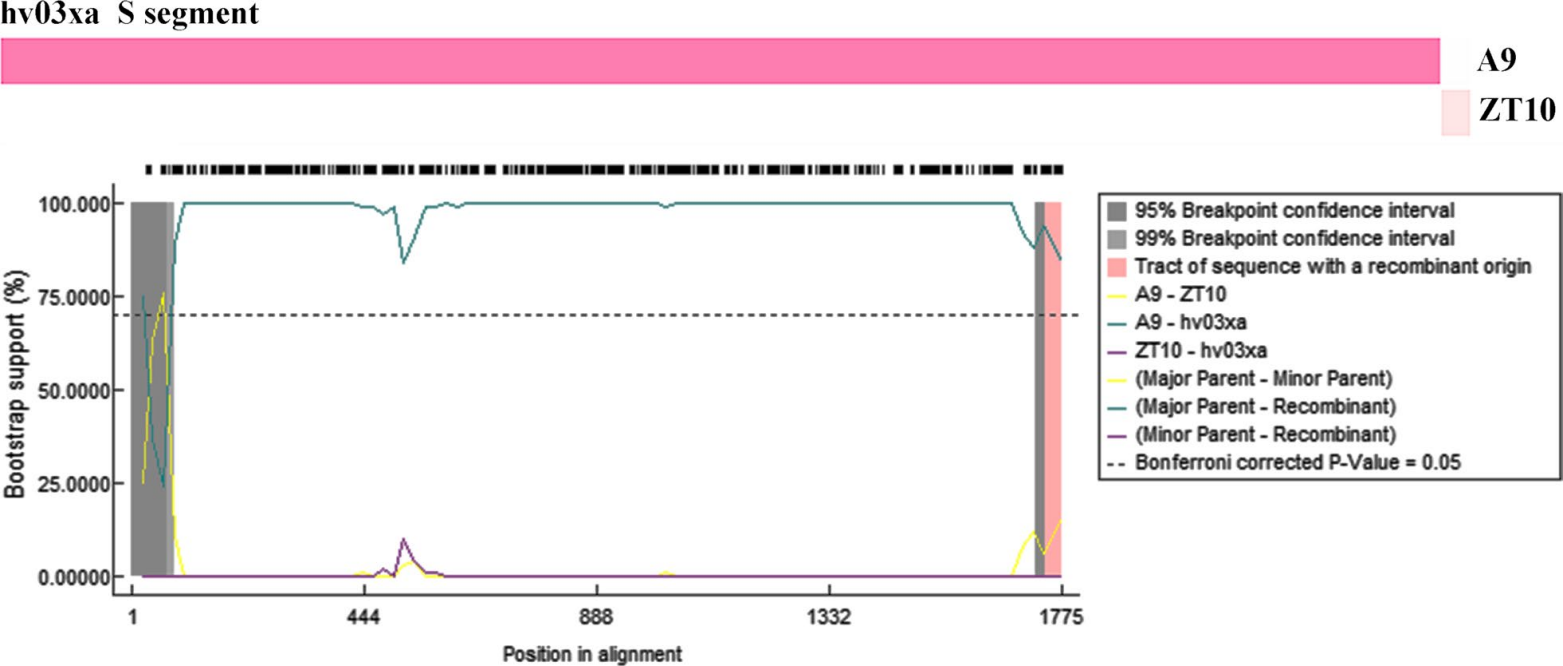
Recombination analysis of hv03xa strain. The Bootscan plots of S segment was based on a pairwise distance model by the RDP4 algorithm. A Bootscan Support Percent of over 70% (cutoff value) was considered significant. The relative strains in the recombination analysis were HTNV TJJ16, 76-118/PRO, Z10, KY, CGRn53, A9, CGRn93MP8, A16, 84FLi, YU61, YU62, CGHu3614, CGHu3612, CGAa75, CA09082007, CA10081206, XAHu09066, and SEOV L99, ZT10

Different methods were employed to provide evidence that recombination could occur in HTNV. Potential recombination event was detected (Table 2). The hv03xa strain may have a recombinant S segment, in which nt 1–1664 originate from the Jiangsu strain A9 of HTNV 9, and nt 1665–1695 originates from Seoul virus strain ZT10 isolated from Zhejiang (Fig. 4). P-value of the analyses was from 3.74E-04 to 1.32E-10 and the RDP recombination consensus score was higher than 0.6 (0.679). The result indicated that hv03xa may have a partial S-segment exchange recombination. No recombination events were detected in the M or L segments of hv03xa strain.

**Table 2:** Potential recombination event of hv03xa strain

Recombinant sequence	hv03xa S segment	
Estimated breakpoint positions	In alignment	1744–1774 nt
	In GenBank	1665–1695 nt
Parental sequences	Major	A9
	Minor	ZT10
*P*-Values of different methods	RDP	1.59E-09
	GENECONV	1.32E-10
	BOOTSCAN	3.00E-10
	MaxChi	3.74E-04
	Chimaera	1.45E-05
	SiScan	2.16E-08
	3Seq	3.42E-08

### IgM and IgG antibody response in patients

**Fig. 5:**
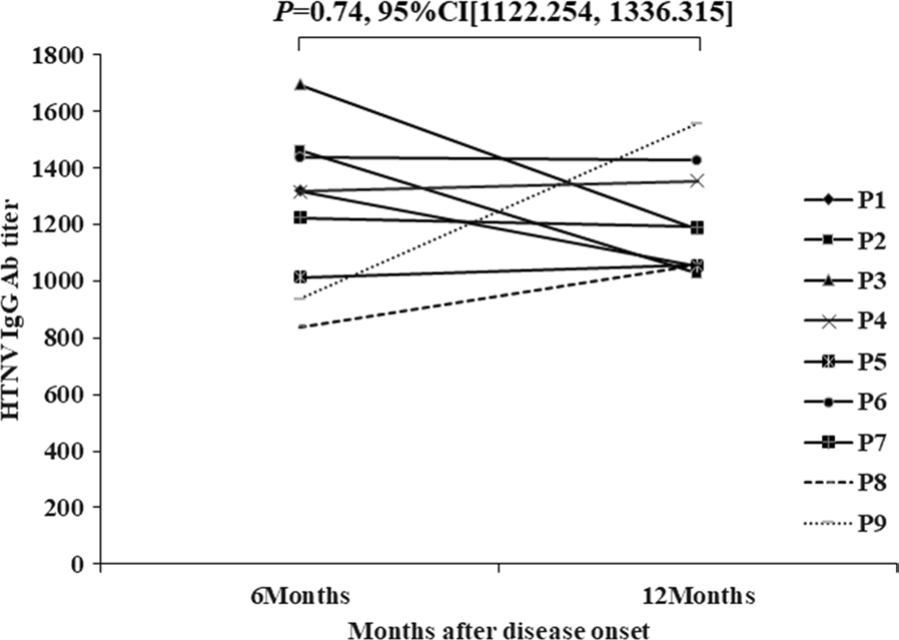
Antibody response to HV in the confirmed 9 patients at 6 months, and 1 year after infection. The p-value and 95% confidence intervals were presented. Each line represented the titer for a patient

The titer above 40 is considered a positive for HTNV-NP-specific IgM and IgG antibodies [9]. Overall, HFRS patients’ serum IgG antibody titers remain detectable and relatively stable one-year PSO (post–symptom onset) (Fig. 5). At 6 months PSO, the geometric mean titer (GMT) was 1248.09 (95% confidence interval [CI] 1067.39–1428.78). At one year after infection, IgG antibody titers were detected in all of 9 patients with a GMT of 1210.48 (95%CI 1085.20–1335.76), which has no significant difference with that of 6 months PSO (*p* < 0.05, Kruskal–Wallis test). The serum IgG antibody titers of patients with HTNV infection was not significantly decreased compared in 1 year later. The HTNV-NP-specific IgM antibody were negative at 6 months and 12 months in all serum samples.

## Discussion

Hantaviruses can survive at room temperature for more than 10 days [35]. Exposure to rodent excrement containing Hantaviruses is the main pathway of human infection with Hantaviruses [35, 36]. The diagnosis of human hantavirus infection can be determined by combining epidemiological and clinical information with laboratory tests. Typical clinical symptoms of HFRS include fever, conjunctival bleeding, abdominal pain, nausea and vomiting, and severe oliguria and acute kidney injury occur in some patients. HFRS patients often have abnormal laboratory markers, such as leukocytosis, thrombocytopenia and elevated serum creatinine and lactate dehydrogenase [11, 12, 37]. In this study, ELISA was used to detect IgM/IgG antibodies of three structural proteins of Hantavirus [18, 19], which suggested that the anti-HV-IgG level of all the patients persist for at least one year after infection.

Among the total nine patients, three took part in carrying cabbages, and four often takes out the rubbish, and two usually went to the tent warehouse. Rats and traces of mouse activity were found in the cabbage tent and the dump, which was considered the most likely risk factors for exposure.

The exchange of genetic components conceals important information, including virus pathogenicity [38]. In this study, nine patients diagnosed with HFRS were most likely acquired HTNV infections from *A. agrarius* positive of HTNV. The genome sequence of the HTNV strain isolated from *A. agrarius* was completely obtained, and phylogenetic analysis indicated that the virus causing this outbreak was probably a reassortment virus, having an S segment related to A16 of HTN 4, an M segment related to Q37 and Q10 of HTN 4, and an L segment related to prototype strain 76–118 of HTN 7 [17].

Recombination events confers genetic diversity in RNA viruses [39]. The genetic events have been observed in Bunyavirus in nature [40]. The exchange of different genetic information is closely related to the function of coding proteins and may result in different recombinants [41]. The exchange of genetic information may be a key factor in the infectivity and pathogenicity of the virus. In this study, possible recombination event was detected in S segment of hv03xa with RDP4, the function of the recombination of S fragment in the pathogenicity of the virus is unknown.

4 of 30 *A. agrarius* captured in the construction site were detected positive for HTNV (data not shown). Avoiding contact with rodents contaminated food and elimination rodents are still important measures to cut off the transmission of hantavirus infection. In the process of urbanization, especially the construction process, more attention should be paid to the destruction of the rodent’s residence and the abnormal density increase of rodents.

These data provided the basic data for epidemiological surveillance of annual endemic HTNV infection and facilitated to predict disease risk and implement prevention measures.

## Supplementary Information

**Additional file 1: Table S1.** The clinical characteristics of the patients.

## Data Availability

The data and materials in this study are freely available. The GenBank accession number for the S, M, L segment of 201120HV03xa reported in this paper is ON661335, OP094683, ON661337, respectively.

## Abbreviations

HFRS: Hemorrhagic fever with renal syndrome
hv03xa: 201120HV03xa
*A. agrarius*: *Apodemus agrarius*

## References

[1] Puca E, Harxhi A, Pipero P, Qyra E, Stroni G, Muco E, Hysenaj Z, Rista E, Tosku G, Bino S (2017). Pancreatitis in patients with hemorrhagic fever with renal syndrome: a five-year experience. J Infect Dev Ctries.

[2] Yu H, Jiang W, Du H, Xing Y, Bai G, Zhang Y, Li Y, Jiang H, Zhang Y, Wang J (2014). Involvement of the Akt/NF-kappaB pathways in the HTNV-mediated increase of IL-6, CCL5, ICAM-1, and VCAM-1 in HUVECs. PLoS ONE.

[3] Klempa B (2009). Hantaviruses and climate change. Clin Microbiol Infect.

[4] Tian HY, Yu PB, Luis AD, Bi P, Cazelles B, Laine M, Huang SQ, Ma CF, Zhou S, Wei J (2015). Changes in rodent abundance and weather conditions potentially drive hemorrhagic fever with renal syndrome outbreaks in Xi’an, China, 2005–2012. PLoS Negl Trop Dis.

[5] Yang X, Wang C, Wu L, Jiang X, Zhang S, Jing F (2019). Hemorrhagic fever with renal syndrome with secondary hemophagocytic lymphohistiocytosis in West China: a case report. BMC Infect Dis.

[6] Zhang S, Wang S, Yin W, Liang M, Li J, Zhang Q, Feng Z, Li D (2014). Epidemic characteristics of hemorrhagic fever with renal syndrome in China, 2006–2012. BMC Infect Dis.

[7] Ma C, Yu P, Nawaz M, Zuo S, Jin T, Li Y, Li J, Li H, Xu J (2012). Hantaviruses in rodents and humans, Xi’an, PR China. J Gen Virol.

[8] Yu PB, Tian HY, Ma CF, Ma CA, Wei J, Lu XL, Wang Z, Zhou S, Li S, Dong JH (2015). Hantavirus infection in rodents and haemorrhagic fever with renal syndrome in Shaanxi province, China, 1984–2012. Epidemiol Infect.

[9] Li Z, Zeng H, Wang Y, Zhang Y, Cheng L, Zhang F, Lei Y, Jin B, Ma Y, Chen L (2017). The assessment of Hantaan virus-specific antibody responses after the immunization program for hemorrhagic fever with renal syndrome in northwest China. Hum Vaccin Immunother.

[10] Du H, Wang PZ, Li J, Bai L, Li H, Yu HT, Jiang W, Zhang Y, Wang JN, Bai XF (2014). Clinical characteristics and outcomes in critical patients with hemorrhagic fever with renal syndrome. BMC Infect Dis.

[11] Qiu FQ, Li CC, Zhou JY (2020). Hemorrhagic fever with renal syndrome complicated with aortic dissection: a case report. World J Clin Cases.

[12] Jonsson CB, Figueiredo LT, Vapalahti O (2010). A global perspective on hantavirus ecology, epidemiology, and disease. Clin Microbiol Rev.

[13] Klingström J, Hardestam J, Lundkvist Å (2006). Dobrava, but not Saaremaa, hantavirus is lethal and induces nitric oxide production in suckling mice. Microbes Infect.

[14] Plyusnin A, Vapalahti O, Vaheri A (1996). Hantaviruses: genome structure, expression and evolution. J Gen Virol.

[15] Lee HW, Lee PW, Johnson KM (2004). Isolation of the etiologic agent of Korean hemorrhagic fever. J Infect Dis.

[16] Kim JA, Kim WK, No JS, Lee SH, Lee SY, Kim JH, Kho JH, Lee D, Song DH, Gu SH (2016). Genetic diversity and reassortment of hantaan virus tripartite RNA genomes in nature, the Republic of Korea. PLoS Negl Trop Dis.

[17] Wang H, Yoshimatsu K, Ebihara H, Ogino M, Araki K, Kariwa H, Wang Z, Luo Z, Li D, Hang C (2000). Genetic diversity of hantaviruses isolated in china and characterization of novel hantaviruses isolated from Niviventer confucianus and Rattus rattus. Virology.

[18] Padula PJ, Rossi CM, Valle MOD, Martinez PV, Colavecchia SB, Edelstein A, Miguel SDL, Rabinovich RD, Segura EL (2000). Development and evaluation of a solid-phase enzyme immunoassay based on Andes hantavirus recombinant nucleoprotein. J Med Microbiol.

[19] Nunes BTD, de Mendonca MHR, Simith DB, Moraes AF, Cardoso CC, Prazeres ITE, de Aquino AA, Santos A, Queiroz ALN, Rodrigues DSG (2019). Development of RT-qPCR and semi-nested RT-PCR assays for molecular diagnosis of hantavirus pulmonary syndrome. PLoS Negl Trop Dis.

[20] Song DH, Kim WK, Gu SH, Lee D, Kim JA, No JS, Lee SH, Wiley MR, Palacios G, Song JW (2017). Sequence-independent, single-primer amplification next-generation sequencing of Hantaan virus cell culture-based isolates. Am J Trop Med Hyg.

[21] Hagele S, Muller A, Nusshag C, Reiser J, Zeier M, Krautkramer E (2019). Virus- and cell type-specific effects in orthohantavirus infection. Virus Res.

[22] Ryou J, Lee HI, Yoo YJ, Noh YT, Yun SM, Kim SY, Shin EH, Han MG, Ju YR (2011). Prevalence of hantavirus infection in wild rodents from five provinces in Korea, 2007. J Wildl Dis.

[23] Zhong XY, Holzgreve W, Huang DJ (2008). Isolation of cell-free RNA from maternal plasma. Methods Mol Biol.

[24] Bolognini D, Bartalucci N, Mingrino A, Vannucchi AM, Magi A (2019). NanoR: a user-friendly R package to analyze and compare nanopore sequencing data. PLoS ONE.

[25] Knyazev A, Glushkevich A, Fesenko I (2020). Direct RNA sequencing dataset of SMG1 KO mutant Physcomitrella (Physcomitrium patens). Data Brief.

[26] Kumar S, Stecher G, Tamura K (2016). MEGA7: molecular evolutionary genetics analysis version 7.0 for bigger datasets. Mol Biol Evol.

[27] Martin DP, Murrell B, Golden M, Khoosal A, Muhire B (2015). RDP4: Detection and analysis of recombination patterns in virus genomes. Virus Evol.

[28] Martin D, Rybicki E (2000). RDP: detection of recombination amongst aligned sequences. Bioinformatics.

[29] Padidam M, Sawyer S, Fauquet CM (1999). Possible emergence of new geminiviruses by frequent recombination. Virology.

[30] Martin DP, Williamson C, Posada D (2005). RDP2: recombination detection and analysis from sequence alignments. Bioinformatics.

[31] Bay RA, Bielawski JP (2011). Recombination detection under evolutionary scenarios relevant to functional divergence. J Mol Evol.

[32] Gibbs MJ, Armstrong JS, Gibbs AJ (2000). Sister-scanning: a Monte Carlo procedure for assessing signals in recombinant sequences. Bioinformatics.

[33] Boni MF, Posada D, Feldman MW (2007). An exact nonparametric method for inferring mosaic structure in sequence triplets. Genetics.

[34] Zhou Z, Deng F, Han N, Wang H, Sun S, Zhang Y, Hu Z, Rayner S (2013). Reassortment and migration analysis of Crimean-Congo haemorrhagic fever virus. J Gen Virol.

[35] Schmaljohn CS, Hasty SE, Dalrymple JM, LeDuc JW, Lee HW, von Bonsdorff CH, Brummer-Korvenkontio M, Vaheri A, Tsai TF, Regnery HL (1985). Antigenic and genetic properties of viruses linked to hemorrhagic fever with renal syndrome. Science.

[36] Clement J, LeDuc JW, Lloyd G, Reynes JM, McElhinney L, Van Ranst M, Lee HW (2019). Wild rats, laboratory rats, pet rats: global Seoul hantavirus disease revisited. Viruses.

[37] Avsic-Zupanc T, Saksida A, Korva M (2019). Hantavirus infections. Clin Microbiol Infect.

[38] Plyusnina A, Plyusnin A (2005). Recombinant Tula hantavirus shows reduced fitness but is able to survive in the presence of a parental virus: analysis of consecutive passages in a cell culture. Virol J.

[39] Simon-Loriere E, Holmes EC (2011). Why do RNA viruses recombine?. Nat Rev Microbiol.

[40] Song JW, Baek LJ, Kim SH, Kho EY, Kim JH, Yanagihara R, Song KJ (2000). Genetic diversity of Apodemus agrarius-borne hantaan virus in Korea. Virus Genes.

[41] Song JW, Baek LJ, Gajdusek DC, Yanagihara R, Gavrilovskaya I, Luft BJ, Mackow ER, Hjelle B (1994). Isolation of pathogenic hantavirus from white-footed mouse (Peromyscus leucopus). Lancet.

