# Correction to: Hemorrhagic fever with renal syndrome caused by destruction of residential area of rodent in a construction site: epidemiological investigation

**DOI:** 10.1186/s12879-022-07767-8

**Published:** 2022-11-30

**Authors:** Xiao Wei, Biao Meng, Hong Peng, Yan Li, Min Liu, Hairui Si, Rui Wu, Hailong Chen, Ying Bai, Qunling Feng, Changjun Wang, Xiangna Zhao

**Affiliations:** 1 Centers for Disease Control and Prevention of PLA, Beijing, China; 2 Department of Epidemiology and Biostatistics, School of Public Health, Anhui Medical University, Hefei, China; 3 PLA 63750 Military Hospital, Xi’an, Shaanxi China; 4 Xi’an Center for Disease Control and Prevention, Xi’an, Shaanxi China

**Correction to**: ***BMC Infect Dis*22, 761 (2022).**10.1186/s12879-022-07744-1

In the original publication there was an incorrect ethics committee. The incorrect and correct information is available in this correction article, the original article has been updated. The other information remains correct (approval code).

## **Incorrect Ethics committee**

-Academy of military medical sciences

## **Correct Ethics committee**

-PLA 63750 Military Hospital

