# Supplementary material for: Hemorrhagic fever with renal syndrome caused by destruction of residential area of rodent in a construction site: epidemiological investigation
Source: BMC Infect Dis. 2022 Sep 29;22:761. doi: 10.1186/s12879-022-07744-1 (PMC9521858; doi:10.1186/s12879-022-07744-1)
Supplement: Supplementary file 1 — Additional file 1: Table S1. The clinical characteristics of the patients. [file 12879_2022_7744_MOESM1_ESM.docx]

**Table S1. The clinical characteristics of the nine patients in this study.**

| **Characteristics** | **P1** | **P2** | **P3** | **P4** | **P5** | **P6** | **P7** | **P8** | **P9** |
| --- | --- | --- | --- | --- | --- | --- | --- | --- | --- |
| Age(years) | 22 | 21 | 23 | 23 | 25 | 30 | 30 | 28 | 27 |
| Sex | Male | Male | Male | Male | Male | Male | Male | Male | Male |
| **Presenting symptoms and signs** |  |  |  |  |  |  |  |  |  |
| Fever | (+) | (+) | (+) | (+) | （+） | (+) | （+） | （+） | （+） |
| Headache | (-) | （+） | (+) | (+) | （+） | (+) | （+） | (-) | （+） |
| Rigor | (-) | (-) | (-) | (+) | （+） | (+) | (-) | （+） | (-) |
| Weak | (-) | (-) | (+) | (+) | （+） | (+) | (-) | (-) | (-) |
| Dizzy | (+) | (-) | (-) | (-) | (-) | (-) | (-) | (-) | （+） |
| Eye pain | (+) | （+） | (-) | (-) | (-) | (+) | （-） | (-) | (-) |
| Nausea | （-） | (-) | (+) | (-) | (-) | (-) | （-） | (-) | （+） |
| Vomiting | (+) | (-) | (-) | (-) | (-) | (-) | (-) | (-) | （+） |
| Abdominal discomfort | (-) | (-) | (-) | (-) | （+） | (-) | (-) | (-) | (-) |
| Diarrhea | (-) | (-) | (-) | (-) | （+） | (-) | （+） | (-) | （+） |
| Cough | (-) | (-) | (-) | (-) | (-) | (-) | (-) | (-) | (-) |
| Dorsalgia | (-) | (-) | (-) | (-) | (-) | (-) | (-) | (-) | (-) |
| Lumbago | (-) | (-) | (-) | (-) | (-) | (-) | (-) | （+） | (-) |
| Pharyngalgia | (-) | (-) | (-) | (-) | (-) | (-) | (-) | （+） | (-) |
| Hypourocrinia | (+) | (-) | (-) | (-) | （+） | （+） | (+) | (-) | (-) |
| Other Diseases | Mild fatty liver | N/A | Gallbladder polyp/Cholecystitis | N/A | Double pneumonia disease | N/A | Fatty liver | N/A | N/A |
| **Blood routine examination** |  |  |  |  |  |  |  |  |  |
| White blood cell (WBC) *10E9/L | 8.69 | 9.45 | 16.79 | 7.98 | 23.83 | 4.63 | 4.77 | 2.71 | 12.18 |
| Percentage of lymphocytes (LYMPH%) | 21.1 | 67.7 | 19.5 | 40.1 | 17.1 | 19.7 | 7.3 | 34.7 | 29.6 |
| Monocyte percentage (MONO%) | 20.7 | 4.7 | 10.8 | 7.1 | 15.7 | 5.8 | 9.9 | 12.9 | 8.4 |
| Percentage of neutrophils (NEUT%) | 56.4 | 25.4 | 68.7 | 50.2 | 66.8 | 72.3 | 81.3 | 51.6 | 61 |
| Eosinophil percentage (EO%) | 1 | 0.7 | 0.8 | 1.8 | 0.3 | 1.1 | 1.3 | 0.4 | 0.7 |
| Absolute value of neutrophil (NEUT#) *10E9/L | 4.9 | 2.4 | 11.53 | 4.01 | 15.92 | 3.35 | 3.88 | 1.4 | 7.42 |
| Absolute value of monocyte (MONO#) *10E9/L | 1.8 | 0.44 | 1.81 | 0.57 | 15.92 | 0.27 | 0.47 | 0.35 | 1.02 |
| Absolute value of lymphocytes (LYMPH#) *10E9/L | 1.83 | 6.4 | 3.28 | 3.2 | 4.08 | 0.91 | 0.35 | 0.94 | 3.61 |
| Absolute value of eosinophils (EO#) | 0.09 | 0.07 | 0.14 | 0.14 | 0.07 | 0.05 | 0.06 | 0.01 | 0.09 |
| Red blood cell (RBC) *10E^9^/L | 3.75 | 4.5 | 6.07 | 3.32 | 4.76 | 5.09 | 5.01 | 4.85 | 5.37 |
| Hemoglobin (HGB g/L) | 111 | 135 | 178 | 106 | 155 | 164 | 144 | 153 | 164 |
| Platelet (PLT) *10E^9^/L | 137 | 199 | 60 | 288 | 41 | 34 | 57 | 87 | 24 |
| **Coagulation function** |  |  |  |  |  |  |  |  |  |
| Prothrombin activity (PTA) | 108 | 101.1 | 83.4 | 70.9 | 89.9 | 83.4 | 77.9 | 74.8 | 67.9 |
| Fibrinogen degradation products (FDP)ug/ml | 19.6 | 3 | 8.9 | 38.7 | 77.24 | 9.9 | 9.1 | 5.86 | 16 |
| D – dimer (D_D) ug/ml | 6.97 | 0.87 | 2.76 | 20.61 | 32.87 | 3.37 | 3.38 | 1.919 | 6.73 |
| Thrombin time (TT) | 18.3 | 19.6 | 29.4 | 22.3 | 106.4 | 28.2 | 27 | 17.4 | 32.9 |
| **Routine urine test** |  |  |  |  |  |  |  |  |  |
| Urine occult blood (BLD) | positive | negative | negative | negative | 3+ | negative | positive | negative | 1+ |
| Urine protein (PRO) | 2+ | negative | 2+ | negative | positive | 1+ | 1+ | negative | 4+ |
| **Stool examination** |  |  |  |  |  |  |  |  |  |
| Occult blood tests（OB) | positive |  | negative |  |  | negative | negative |  |  |
| **Liver function** |  |  |  |  |  |  |  |  |  |
| Alanine aminotransferase (ALT) | 63 | 103 | 65 | 53 | 77 | 46 | 125 | 36 | 100 |
| Aspartate transaminase (AST) | 41 | 52 | 74 | 28 | 205 | 79 | 97 | 53 | 134 |
| Albumin (ALB) | 25.6 | 38.2 | 38.1 | 46.1 | 34.8 | 33.7 | 28.5 | 39.1 | 29.8 |
| Conjugated bilirubin (BC) | 5.14 | 3.65 | 3.58 | 1.9 | 4 | 2.44 | 9.68 | 3.86 | 2.5 |
| Total protein (TP) | 54.6 | 65 | 66.4 | 68.2 | 65 | 59.2 | 58.7 | 68.8 | 55.1 |
| **Renal function** |  |  |  |  |  |  |  |  |  |
| Blood urea nitrogen（BUN）mmol/L | 12.2 | 5.85 | 4.32 | 4.68 | 9.4 | 3.92 | 10.42 | 4.77 | 8.23 |
| Creatinine（Cr)umol/L | 251.3 | 90.5 | 66 | 100 | 145 | 90.2 | 131.1 | 61 | 168.3 |
| Egfr (CKD) n/1.73m2 |  | 104.14 | 129.55 | 91.01 | 57.27 | 98.15 | 62.45 | 129.2 | 47.16 |
| **HTNV-NP-specific IgG antibodies** |  |  |  |  |  |  |  |  |  |
| IgM | （+） | （+） | （+） | （±） | （+） | （±） | （+） | （+） | （+） |
| IgG | （±） | （+） | （+） | （+） | （+） | (-) | （+） | （+） | （+） |
| **Myo-cardial enzymonram** |  |  |  |  |  |  |  |  |  |
| Total cholesterol（TC）mmol/L |  |  |  |  |  |  | 2.58 | 3.33 | 3.24 |
| Triglyceride （TG）mmol/L |  |  |  |  |  |  | 0.91 | 1.81 | 3.09 |
| High density lipoprotein cholesterol（HDL-C) mmol/L |  |  |  |  |  |  | 0.62 | 0.63 | 0.55 |
| Low density lipoprotein cholesterin（LDL-C) mmol/L |  |  |  |  |  |  | 1.51 | 1.95 |  |
| Creatine kinase（CK） U/L | 62 | 30 |  | 92 |  |  | 107 | 34 | 163 |
| Creatine kinase-MB（CK-MB） U/L | 6 | 8 |  | 14 |  |  | 15 | 15 | 22 |
| Lactic dehydrogenase（LDH） U/L | 324 | 321 |  | 376 |  |  | 355 | 326 | 503 |
| α-Hydroxybutyrate Dehydrogenase（α-HBDH）U/L | 289 | 261 |  | 276 |  |  | 266 | 242 | 324 |
| Classification of hemorrhagic fever with renal syndrome | Moderate | Mild | Moderate | Severe | Moderate | Moderate | Severe | Mild | Severe |
